# The efficacy and safety of an adapted opioid-free anesthesia regimen versus conventional general anesthesia in gynecological surgery for low-resource settings: a randomized pilot study

**DOI:** 10.1186/s12871-022-01856-6

**Published:** 2022-10-24

**Authors:** Joel Noutakdie Tochie, Roddy Stephan Bengono Bengono, Junette Mbengono Metogo, Raymond Ndikontar, Serges Ngouatna, Ferdinand Ndom Ntock, Jacqueline Ze Minkande

**Affiliations:** 1 Department of Surgery and Sub-Specialties, Faculty of Medicine and Biomedical Sciences, University of Yaoundé 1, Yaoundé, Cameroon; 2 Department of Anesthesiology and Critical Care Medicine, Sangmelima Reference Hospital, Sangmelima, Cameroon; 3 Department of Surgery and Sub-Specialties, Faculty of Medicine and Pharmaceutical Sciences, University of Douala, Douala, Cameroon; 4 Department of Anesthesiology and Critical Care Medicine, Douala General Hospital, Douala, Cameroon; 5 Department of Anesthesiology and Critical Care Medicine, Yaoundé Gyneco-Obstetric and Pediatric Hospital, Yaoundé, Cameroon; 6 Department of Anesthesiology and Critical Care Medicine, Yaoundé Emergency Center, Yaoundé, Cameroon

**Keywords:** Opioid-free anesthesia, Adapted, Gynecological surgery, Efficacy, Safety, Cameroon

## Abstract

**Introduction:**

There is scarce data on the safety and efficacy of opioid-free anesthesia (OFA), in resource-limited settings due to the non-availability of dexmedetomidine, the reference OFA agent. We aimed to demonstrate the feasibility, efficacy and safety of a practical OFA protocol not containing dexmedetomidine, adapted for low-resource environments in very painful surgeries like gynecological surgery.

**Methods:**

We conducted a randomized pilot study on ASA I and II women undergoing elective gynecological surgery at a tertiary care hospital in Cameroon. Patients were matched in a ratio of 1:1 into an OFA and a conventional general anesthesia (CGA) group. The OFA protocol entailed the intravenous (IV) magnesium sulfate, lidocaine, ketamine, dexamethasone, propofol, and rocuronium, followed by isoflurane and a continuous infusion of a calibrated mixture of magnesium sulfate, ketamine and clonidine. The CGA protocol was IV dexamethasone, diazepam, fentanyl, propofol, and rocuronium, followed by isoflurane and reinjections of fentanyl propofol and a continuous infusion of normal saline as placebo. The primary endpoints were the success rate of OFA, isoflurane consumption and intraoperative anesthetic complications. The secondary endpoints were postoperative pain intensity, postoperative complications, patient satisfaction assessed using the QoR-40 questionnaire and the financial cost of anesthesia.

**Results:**

We enrolled a total of 36 women undergoing gynecological surgery; 18 in the OFA group and 18 in the CGA group. The success rate of OFA was 100% with significant lesser consumption of isoflurane in the OFA group, no significant intraoperative complication and better intraoperative hemodynamic stability in the OFA group. Postoperatively, compared to the CGA group, the OFA group had statistically significantly less pain during the first 24 h, no morphine consumption for pain relief, had less hypoxemia during the first six hours, less paralytic ileus, less nausea and vomiting, no pruritus and better satisfaction. The mean financial cost of this adapted OFA protocol was statistically significant lesser than that of CGA.

**Conclusion:**

This OFA regimen without dexmedetomidine for a low-resource setting has a promising success rate with few perioperative complications including mild intraoperative hemodynamic changes, decrease postoperative complications, pain, and opioid consumption in patients undergoing elective gynecology surgery.

**Trial registration:**

This study was registered at clinicaltrials.gov on 03/02/2021 under the registration number NCT04737473.

**Supplementary Information:**

The online version contains supplementary material available at 10.1186/s12871-022-01856-6.

## Introduction

For the last six decades, opioids have been a key component of conventional general anesthesia (CGA) due to their analgesic effect, their hypnotic effect and their ability to control the autonomic nervous system response to surgical stress during CGA, with resultant hemodynamic stability [1, 2]. However, recently, the principle underlying the administration of opioids during CGA has been subjected to several opioid-related adverse effects like postoperative respiratory depression [3], postoperative ileus (POI), postoperative nausea and vomiting (PONV) [4], hyperalgesia [5], inflammation modulation, immune depression especially in oncology surgery [6], poor postoperative analgesia, increased consumption of morphine [7], pruritus, urinary retention [8] and postoperative shivering [9]. These lead to undesirable delayed patient rehabilitation, prolonged length of hospital stay (LOS), increase cost of healthcare, poor patient satisfaction, not accepted in the current era of enhanced recovery after surgery (ERAS) [10]. In a bid to mitigate the aforementioned opioid-related adverse effects in the perioperative period, an opioid-sparing anesthetic technique called Opioid-Free Anesthesia (OFA) was recently developed [11]. OFA is a technique of providing anesthesia by combining intravenous administration of a combination of non-opioid agents to produce adequate intraoperative analgesia, hypnosis, sympatholysis, and pain-free awakening [12–14]. Some small sample-size clinical studies limited to bariatric surgery and laparoscopic cholecystectomy have fairly demonstrated the superiority of OFA over CGA in procuring several benefits such as better intraoperative analgesia, hypnosis, myorelaxation, hemodynamics, bispectral index [14–16] with a reduction in postoperative pain [9, 15, 17], postoperative opioid consumption [9, 17, 18], PONV [9, 17, 19], postoperative hypoxemia [9], higher postoperative patient satisfaction [9, 20] and shorter length of stay (LOS) in the postanesthesia care unit (PACU) [20].

Gynecological surgery, including breast cancer surgery, leads to intense surgical stress, making gynecological surgery particularly prone to intense postoperative pain, inflammation, marked shivering, severe PONV and POI [21]. More specifically, following major gynecological surgery, PONV occur at incidences of 50—80% [22, 23] and severe acute and chronic postoperative at 40% [24]. Hence, the aforementioned benefits of OFA, make this anesthetic technique an attractive promising option for gynecological surgery [21]. However, the extent of the aforementioned benefits of OFA is questionable because dexmedetomidine, the reference OFA analgesic-sedative drug, recently used in a well-powered multicenter clinical trial on 314 non-cardiac surgeries (including 18 gynecological surgeries), was associated with more statistically significant delayed extubation, prolonged PACU LOS, postoperative hypoxemia, and severe bradycardia warranting early study termination [25]. Two other large well-powered recent studies [26, 27] conducted in high-income settings, comparing OFA (using dexmedetomidine) to CGA in laparoscopic gynecological surgery showed equivalent postoperative pain, postoperative opioid consumption, PONV, anti-emetic requirements [26, 27] with even more significant sedation scores and longer PACU LOS in the OFA group [27]. On the other hand, studies [28–31] carried out in low-and middle-income countries above the Saharan region with some using dexmedetomidine in OFA for gynecological surgery are in favour of better hemodynamic stability in addition to the aforementioned benefits of OFA. With an extensive literature search, to the best of our knowledge, there is a dearth of comprehensive or reference data on the feasibility, safety and effectiveness of OFA in gynecological surgery in sub-Saharan Africa, the most poverty stricken region of the world and where dexmedetomidine is not available due to its relatively high financial cost. Hence, we propose this study to report on the feasibility, safety and efficacy of an adapted OFA protocol not containing dexmedetomidine in women undergoing gynecological surgery in a major referral Mother and Child Hospital in Cameroon.

## Materials and methods

This manuscript adheres to the applicable 2010 CONSORT guidelines for randomized controlled trials (supplementary file 1).

### Ethical considerations

Before the start of the study, the study protocol was approved by the Institutional Review Board of the Faculty of Medicine and Biomedical Sciences, University of Yaoundé I, Yaoundé, Cameroon (approval number: 223/UYI/FMSB/VDRC/DAASR/CSD). This clinical trial was registered at ClinicalTrials.gov; NCT04737473; February 03, 2021; principal investigator: Tochie JN (https://clinicaltrials.gov/ct2/show/NCT04737473)*.*

### Study design and study setting

This was a randomized controlled trial carried out at the Department of Anesthesiology and Intensive Care of the Yaoundé Gyneco-obstetric and Pediatric Hospital (YGOPH), Yaoundé, Cameroon between January 06, 2020, to September 28, 2021 (21 months). The YGOPH is a tertiary and University Teaching Hospital for the referral of mother and child illness in Yaoundé and its environs. The study was carried out precisely at the Anesthesiology unit and Intensive Care Unit (ICU) of the YGOPH is run by four attending Anesthesiologist-Intensive Care Physicians, 13 anesthesiologist-intensive care nurses and 16 state registered nurses.

### Participant eligibility and sample size calculation

Eligible participants were adult non-pregnant women aged** ≥ **18 years classified American Society of Anesthesiology (ASA) I and II, admitted to the study setting for an elective myomectomy, hysterectomy, ovarian cystectomy or total mastectomy for benign pathologies and localized malignancies and who were able to follow instructions and comply with assessments. The exclusion criteria were history of allergy to any drug used for OFA or CGA; history of alcohol, opioid or drug abuse; chronic pain; psychiatric illness; patients undergoing surgery with planned regional anesthesia of tissular infiltration of local anesthesia, those with iatrogenic surgical complications such as bowel, ureter or bladder injuries. The sample size was estimated using the formula: *n* = 2(Zα + Z[1-β])^2^ × P × q/d^2^ [32]. Zα is the standard normal variate equal to 1.96, the power was set at 80%, the level of significance (α) at 5%, the effect size is d and q is 1—P. P is the pre-study estimate of the prevalence of gynecology surgery like hysterectomy at the Douala General Hospital of Cameroon which is 14.54% [33]. A reduction of postoperative pain by 40% will be considered significant for the effectiveness of the adapted OFA protocol. Hence, the minimum sample size calculated was 34 patients; we needed a minimum of 17 in the CGA group and 17 in the OFA group.

### Participants’ randomization and blinding

Patient enrolment was done at the outpatient Anesthesiology unit solely by the principal investigator who recruited all adult women ASA I and II who signed informed consent for an elective myomectomy, hysterectomy, ovarian cystectomy or total mastectomy. Patients were matched for age, parity, comorbidities, type of gynecological surgical procedure and ASA grade in a ratio of 1:1 to the OFA group and CGA group. Simple randomization was done by consecutive enrolment and encoding identifications of OFA cases on Mondays and Wednesdays, followed by CGA cases on Tuesdays and Thursdays, until attainment of the minimum sample size. This randomization, determined by surgical days of the week was chosen by the principal investigator solely for this study and only the principal investigator knew the encoding concealment. At induction, only the principal investigator aided by a certified experienced external anesthesiologist and intensive care physician not involved in patient care were present in the operating room and knew whether the patient was administered OFA or CGA. Thereafter, the certified experienced external anesthesiologist and intensive care physician not involved in patient care went out of the operating room. The surgical team and the rest of the anesthetist team both entered the operating room when the patient was administered maintenance anesthesia via an unlabeled infusion of either opioid-free analgesics (for the OFA group) or normal saline (for the CGA group). Apart from the principal investigator (JNT) and the certified experienced external anesthesiologist and intensive care physician not involved in patient care, patients, surgeons and the rest of the anesthetist and ICU teams were thus blinded to the OFA or CGA allocation.

### Anesthetic management

On arrival at the theater, all patients received standard monitoring including ECG, pulse oximetry, non-invasive blood pressure and temperature. Thereafter, we began compensating any un-prescribed or undue clear oral fluid fasting by the patient for more than two hours with Ringer Lactate Solution, followed by antibiotic prophylaxis with intravenous (IV) amoxicillin-clavulanic acid 2 g, and then, pre-oxygenation. The anesthetic protocols are as follows;

OFA was induced using an adapted French protocol by Beloeil H [34]; premedication with lidocaine 1.5 mg/kg IV, magnesium sulfate 40 mg/kg (in 100 ml of saline without exceeding 2.5 g), ketamine 25 mg IV and dexamethasone 0.1 mg/kg IV. Induction of general anesthesia with propofol 1.5 mg/kg IV and rocuronium 0.1 mg/kg IV. Anesthesia was maintained using isoflurane between 0.5–2%, and an electric pump syringe at 10–15 ml/h containing a mixture of magnesium sulfate 40 mg/kg (without exceeding a total dose of 2.5 g/24 h taking note of the induction dose), lidocaine 1.5 mg/kg, ketamine 0.25 mg, and clonidine 1 ug/kg.

CGA protocol consisted of premedication with diazepam 5 mg IV and dexamethasone 0.1 mg/kg IV. The anesthesia was induced using fentanyl 3ug/kg IV, propofol 2.5 mg/kg IV, and rocuronium 0.1 mg/kg IV. Anesthesia was maintained using isoflurane between 0.5–2%, reinjections of one-quarter of the induction dose of fentanyl every 20 – 30 min and one-quarter of the induction dose of propofol as needed and a continuous infusion of normal saline via an electric pump syringe at 10–15 ml/h as placebo.

In both groups after induction of anesthesia, patients were relayed to an anesthesia machine (MINDRAY WATO EX65) where they were volume-controlled ventilated at the following parameters: tidal volume at 6 – 8 ml/kg, respiratory rate at 12 – 14 breaths/min, inspiratory: expiratory fraction at 1: 2, positive end-expiratory pressure at 2 – 4 cmH_2_0, and end-tidal volume of carbon-dioxide (ETCO_2_) at 30–50 mmHg. Intraoperative resuscitation consisted of compensating the fasted fluids, administering hourly fluid maintenance, replenishing fluid losses volume by volume with Ringer Lactate solution; blood transfusion when the amount of blood loss authorized was exceeded; fluid bolus and reduction/cessation of isoflurane in case of hypotension; administering atropine 0.1 mg/kg IV in case of bradycardia; nicardipine 1 – 2 mg bolus against high blood pressure. Approximately 30 min before skin surgical closure, all patients received IV paracetamol 1 g, IV tramadol 100 g and IV nefopam 20 mg.

### Adverse effects

These include any intra-operative undesirable effects of OFA or CGA such as hypotension, high blood pressure, bradycardia, and tachycardia. Hypotension was defined as a systolic blood pressure of less than 90 mmHg or a mean arterial pressure of less than 65 mmHg. High blood pressure was a systolic blood pressure greater than 140 mmHg and/or diastolic blood pressure greater than 90 mmHg. A pulse less than 60 beats per minute was termed bradycardia, whereas, a pulse greater than 99 beats per minute was termed tachycardia. Adverse effects also included post-operative undesirable effects of OFA or CGA such as postoperative hypoxemia and postoperative ileus (POI). Postoperative hypoxemia was defined as therapeutic oxygen supplementation to maintain SpO2 > 95% within the first 48 h after extubation [34]. POI was defined as an absence of flatus or stools within the first 48 h after extubation [34].

### Postoperative phase

Due to the pilot nature of this study and the concern of post-anesthesia security, after surgery, all patients were admitted to the ICU till hospital discharge for close monitoring of any adverse effects of OFA and CGA. Analgesics were administered based on the Numerical Rating scale (NRS), interpreted in Table 1.

**Table 1:** Numerical Rating scale interpretation

Numerical Rating scale (NRS)	Meaning	Management
≤ 3	Mild pain	Paracetamol 1 g/6 h IV
3 – 6	Moderate pain	Paracetamol 1 g/6 h IV, diclofenac 75 mg/12 h IM (for 48 h) and tramadol 100 mg/8 h IV
≥ 7	Severe pain	Paracetamol 1 g/6 h IV, diclofenac 75 mg/12 h IM (for 48 h) and tramadol 100 mg/8 h IV plus morphine at titrated doses

### Outcomes or endpoints

The primary outcomes were the failure of OFA (defined as the intraoperative need to administer opioids for adequate intraoperative analgesia) and the occurrence of intra-operative complications e.g. hypotension, high blood pressure, tachycardia and bradycardia. Other primary endpoints were the mean alveolar concentration (MAC in %) of isoflurane used in both groups.

The secondary endpoints were pain intensity and patient satisfaction within the first 48 h postoperation, as well as the occurrence of postoperative complications until hospital discharge as detailed below: (1) pain intensity (assessed using the Numerical Rating scale) and need for opioids for pain relief with the first 48 h of surgery; (2) patient satisfaction (assessed on the Quality of Recovery-40 [QoR-40] questionnaire in supplementary file 1) at 24 h and 48 h postoperation. The QoR-40 questionnaire is a universally or externally validated scale for the assessment of patient satisfaction after major surgery based on five criteria of recovery: physical comfort, emotional state, physical independence, psychological support, and pain [28, 34]. The QoR-40 questionnaire is graded from 40 to 200, where QoR-40 = 40, QoR-40 between 41 and 159 and QoR-40 ≥ 160 indicate poor, moderate and good patient satisfaction; (3) the occurrence and number of PONV episodes (managed by fluid hydration as well as anti-emetic; dexamethasone 4 mg IV bolus); (4) postoperative hypoxemia; (5) the need of endotracheal re-intubation with mechanical ventilation; (6) POI; (7) time between the end of OFA or CGA maintenance and extubation; (8) hospital length of stay (max. 28 days) defined as the number of days after extubation before first hospital discharge; (9) other secondary endpoints: pruritus, need of oxygen postoperatively, time for the return of intestinal transit, mean time between the end of surgery and the first walk in hours, and mean total cost of drugs used for anesthesia (XAF).

### Follow-up after discharge

Follow-up anesthesia visits at one, two, three and four weeks after hospital discharge for clinical assessment in search of any potential complaint related to OFA or CGA. Any complaint/complication found was treated accordingly.

### Statistical analysis

Descriptive analysis was done using percentages of binary variables, mean (standard deviations) and median (interquartile range) for continuous variables. The primary effect variable: the failure of OFA was analyzed and intraoperative complications were analyzed as dichotomous variables (present/absent). Continuous variable analysis was applied to secondary effect variables such as time between the end of OFA or CGA maintenance and extubation, hospital length of stay, time for the return of intestinal transit, mean time between the end of surgery and the first walk, and mean total cost of drugs used for anesthesia (XAF). Other secondary effect variables such as QoR-40 scale score, PONV, postoperative hypoxemia, need for endotracheal re-intubation, POI, pruritus and need for oxygen postoperatively were analyzed as dichotomous variables (present/absent). The distribution of data was assessed using the Kolmogorov-Smirnoff test. A comparison of categorical data between the OFA and CGA groups was performed using the Chi-square test and Fischer exact test. ANOVA test was performed for repeated measures to assess postoperative pain reported through the NRS at different time intervals (1st, 2^nd^, 6th, 12th, 24th and 48th hour) between the OFA and CGA groups. The threshold of statistical significance was set at 0.05. All statistical analyses were performed using EPI INFO version 3.5.3 software.

## Results

**Fig. 1:**
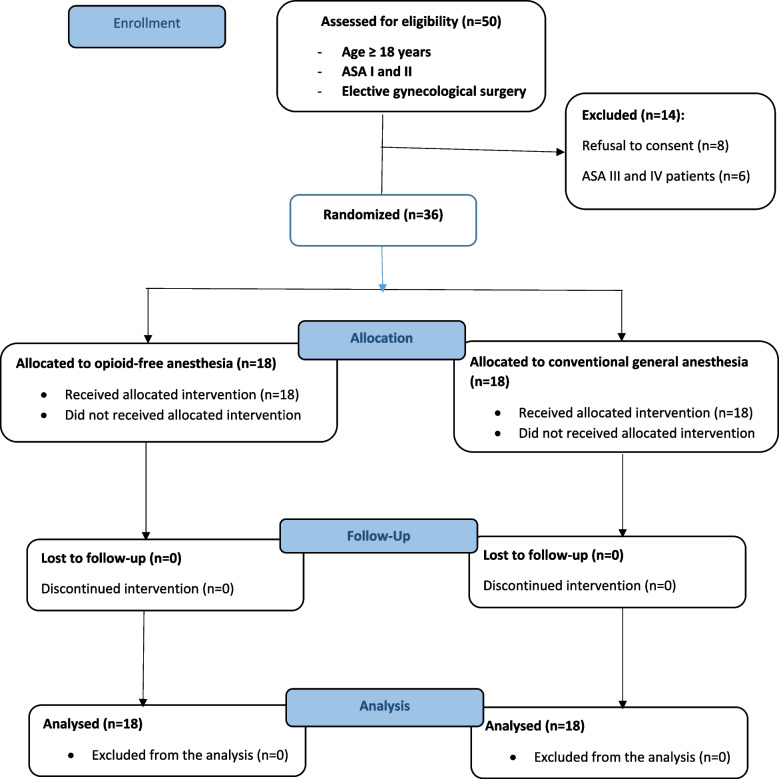
CONSORT 2010 flow diagram illustrating participants’ enrollment

A total of 50 women booked for elective gynecology surgery were approached. Fourteen were excluded: eight refused to consent; six were classified ASA IV due to two patients with a recent ischemic stroke (four months ago), two patients on recent chemotherapy (2 weeks ago) for breast cancer with abnormal hepatic function test and two patients on maintenance hemodialysis for end-stage renal. Hence, 36 participants were retained as the study population, yielding a response rate of 72%. Figure 1 illustrates a flow diagram for participant enrolment.

### Baseline characteristics of the study population

The mean age of the women was 40.3 ± 10.7 years (range: 18 to 63 years). The OFA and CGA group were comparable in terms of mean age, mean parity, age, parity, comorbidities, types and indications of gynecological surgical procedure, mean systolic blood pressure, mean diastolic blood pressure, mean arterial pressure, mean oxygen peripheral saturation and ASA grade (all p above 0.05) (See Table 2).

**Table 2:** Baseline characteristics of the study population

Characteristics	Total sample *N* = 36 (%)	OFA group *N* = 18 (%)	CGA group *N* = 18 (%)	RR	95% CI	*p*—value
**Age (mean in years)**	40.3 ± 10.7	39.2 ± 12.2	41.4 ± 9.2	-	-	0.6227
**Parity**	3.4 ± 2.8	3.1 ± 2.8	3.8 ± 2.8	-	-	0.4233
**Past history**				-	-	0.3456
None	32 (88.8)	15 (83.3)	17 (94.4)			
HIV	2 (5.6)	1 (5.6)	1 (5.6)			
Hypertension	2 (5.6)	2 (11.1)	0 (0)			
**Type of surgery**				-	-	0.0555
Myomectomy	17 (47.2)	5 (27.8)	12 (66.7)			
Ovarian cystectomy	8 (22.2)	7 (38.9)	1 (5.5)			
Hysterectomy	6 (16.7)	3 (16.7)	3 (16.7)			
Mastectomy	5 (13.9)	3 (16.7)	2 (11.1)			
**Surgical Indications**				-	-	0.1811
**Mymectomy**						
Fibroid – menometrorrh	13 (36.1)	6 (33.3)	7 (38.9)			
Fibroid – infertility	4 (11.1)	1 (5.6)	3 (16.7)			
Fibroid—pain	1 (2.8)	0 (0)	1 (5.6)			
**Hysterectomy**						
Localized cervical cancer	4 (11.1)	1 (5.6)	3 (16.7)			
**Mastectomy**						
Invasive ductal carcinoma	6 (16.7)	3 (16.7)	3 (16.7)			
**Ovarian cystectomy**						
Benign ovarian cyst	8 (22.2)	7 (38.9)	1 (5.6)			
**Pre-anaesthesia parameters**						
Mean SBP (mmHg)	124.8 ± 12.8	122 ± 14.1	127.7 ± 11.1	-	-	0.1525
Median SBP (mmHg)	126	119.5	126	-	-	0.1525
Mean DBP (mmHg)	77.4 ± 10.8	76 ± 11.4	78.8 ± 10.2	-	-	0.4957
Median DBP (mmHg)	80	78.5	81	-	-	0.4957
Mean of MAP (mmHg)	89 ± 29.9	82.8 ± 40.9	95.3 ± 9.1	-	-	0.2168
Median MAP (mmHg)	93.5	93	96.5	-	-	0.2168
Mean pulse (bpm)	84.6 ± 13.4	82.9 ± 15.1	86.2 ± 11.7	-	-	0.3110
Median pulse (bpm)	83	78.5	87	-	-	0.3110
Mean SaO_2_ (%)	97.4 ± 14.9	99.8 ± 0.5	95.0 ± 21.2	-	-	0.5975
Median SaO_2_ (%)	100	100	100	-	-	0.5975
**Mean BMI (kg/m**^**2**^**)**	25.7 ± 5.1	23.9 ± 5.7	27.5 ± 3.6	3.6	0.37 – 6.83	0.0080
**ASA**				0.7	0.37 – 1.33	0.2443
I	23 (63.8)	13 (72.2)	10 (55.6)			
II	13 (36.1)	5 (27.8)	8 (44.4)			

*ASA* American Society of Anesthesiology, *bpm* Beats per minute, *BMI* Body mass index, *CGA* Conventional general anesthesia, *CI* Confidence interval, *DPB* Diastolic blood pressure, *MAP* Mean arterial pressure, *menometrorrh* Menometrorrhagia, *OFA* Opioid-free anesthesia, *RR* RR ratio, *SaO*_*2*_ Oxygen peripheral saturation, *SBP* Systolic blood pressure

### Primary endpoints

**Fig. 2:**
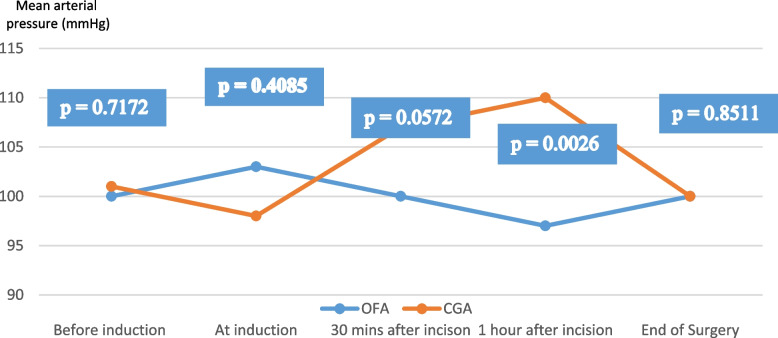
Means of mean arterial pressures intraoperative variations between the OFA and CGA groups

**Fig. 3:**
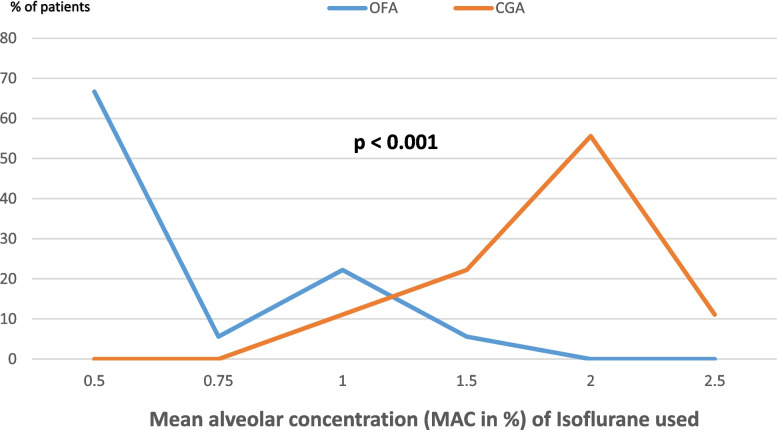
Intraoperative consumption of halogen gazes in the OFA and CGA groups

The success rate of OFA was 100% and no intraoperative complication was observed in the OFA group (Table 3). The intraoperative hemodynamics mainly evaluated using the variation in the mean arterial pressure (MAP) showed more stable hemodynamics in the OFA group compared with the CGA group. Moreover, the MAP at one hour after the surgical incision was significantly more stable in the OFA group than in the CGA group (*p* = 0.0026) as shown in Fig. 2. Isoflurane, the halogen gas partly used for maintenance anesthesia in both the OFA and CGA groups was statistically consumed less in the OFA group compared with the CGA group (*p* < 0.001) (See Fig. 3).

**Table 3:** Intraoperative complications

Intraoperative complication	OFA group *N* = 18	CGA group *N* = 18	RR	95% CI	*P* value
Bradycardia	0	0	-	-	0.5000
Hypotension	0	1	-	-	0.5000
Tachycardia	0	4	-	-	0.5000
High blood pressure	0	0	-		0.5000

*CI* Confidence interval, *OR* Risk ratio

### Secondary endpoints

**Fig. 4:**
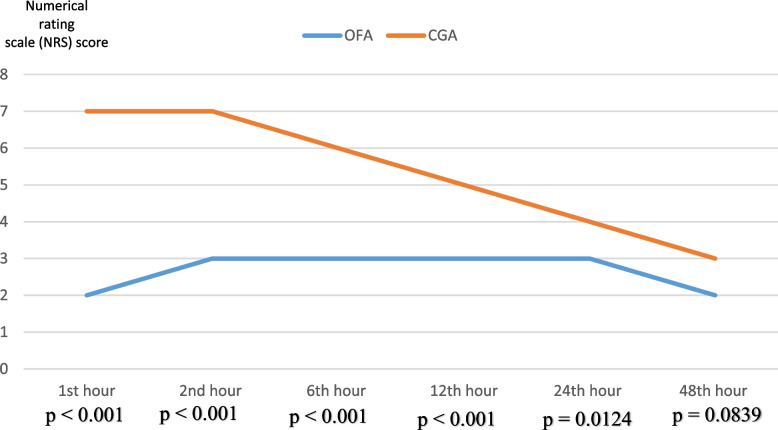
Postoperative pain intensity variations in the OFA and CGA groups. NRS = 7 at 1^st^ hour occurred only in mastectomies and hysterectomies. NRS was statistically different at 12 h and 24 h mainly in mastectomies

**Fig. 5:**
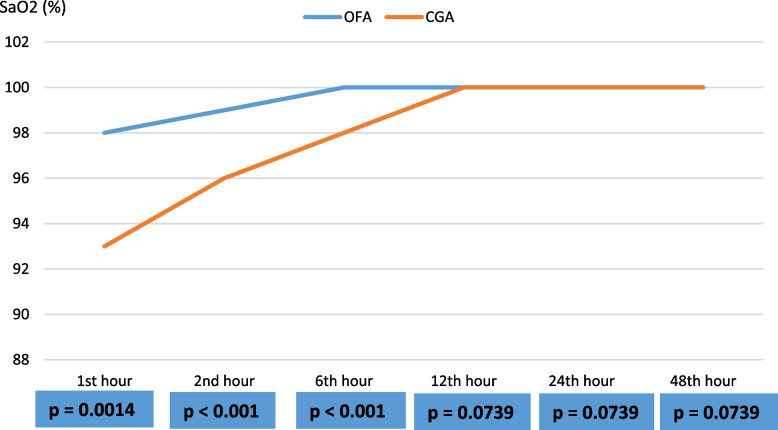
Means of postoperative peripheral oxygen saturation variations between the OFA and CGA groups

Patients who underwent OFA were extubated faster than those who underwent CGA though statistically insignificant. Also, compared to the CGA group, the OFA group had lesser statistically significant pain scores within the first 24 h post-operation (Fig. 4) and did not require morphine for postoperative pain relief; had fewer hypoxemic episodes within the first six-hour post-operation (Fig. 5) and required lesser oxygen postoperatively; had no POI; had lesser episodes of PONV; had no pruritus; rehabilitated or began walking post-operatively faster after surgery; had good postoperative satisfaction compared with moderate postoperative satisfaction in the OFA group (all *p* < *0.05*). The mean financial cost of OFA was statistically significantly lesser than the mean cost of CGA (46,500 ± 2,573 XAF *vs.* 57,000 ± 5,379 XAF; *p* < 0.001) (See Table 4).

**Table 4:** Secondary endpoints

Variable	OFA group *N* = 18 (%)	CGA group *N* = 18 (%)	RR	95% CI	*p* – value
Mean time between the end of maintenance anesthesia and extubation in minutes (SD)	13.61 ± 6.55	17.67 ± 6.25	-	-	0.0705
Median time between the end of maintenance anesthesia and extubation in minutes	11	18.5	-	-	0.0705
Need of morphine for postoperative pain relief	0	6	2.5	1.61–3.89	0.0095
Need of oxygen postoperatively	1 (5.6%)	12 (66.7%)	12	1.74–82.89	< 0.001
Need of reintubation	0 (0%)	0 (0%)	-	-	-
Postoperative ileus	1 (5.6%)	15 (83.3%)	15	2.21–101.9	< 0.001
Mean hours for return of intestinal transit (SD)	15.7 ± 5.5	71 ± 25	55.3	43.04–67.56	< 0.001
Median hours for return of intestinal transit	17	74	-	-	< 0.001
Postoperative nausea and vomiting	2 (11.1%)	9 (50%)	4.5	1.13–17.99	0.0137
Mean hour of occurrence of PONV (SD)	2.0 ± 0.0	1.6 ± 0.69	-	-	0.3961
Mean hour of occurrence of PONV	2.0	2.0	-	-	0.3961
Pruritus	0 (0%)	6 (33.3%)	-	-	0.0095
Mean time between the end of surgery and the first walk in hours (SD)	10.44 ± 3.60	21.94 ± 9.48	11.5	6.64 – 16.36	< 0.001
Median time between the end of surgery and the first walk in hours	10	24	-	-	< 0.001
Postoperative satisfaction at the 48^th^ hour (mean QoR-40 score)	179.72 ± 8.70 (good satisfaction)	84.11 ± 42.71 (Moderate satisfaction)	-	-	< 0.001
Mean length of hospital stay in days (SD)	5.06 ± 0.42	5.22 ± 0.65	-	-	0.4328
Median length of hospital stay in days	5	5	-	-	0.4328
Mean total cost of drugs used for anesthesia (XAF)	46,500 ± 2,573	57,000 ± 5,379	-	-	< 0.001

*CGA* Conventional general anesthesia, *CI* Confidence interval, *PONV* Postoperative nausea and vomiting, *OFA* Opioid-free anesthesia, *RR* Risk ratio, *SaO2* Oxygen peripheral saturation, *QoR-40* Quality of Rehabilitation, *SD* Standard deviation

### Follow-up after discharge

Patients in both the adapted OFA and CGA groups were followed up till four weeks after anesthesia with no complications in both groups.

## Discussion

This study showed that the proposed OFA technique without dexmedetomidine has a promising success rate with few perioperative complications including mild intraoperative hemodynamic changes, decrease postoperative complications, pain, and opioid consumption in patients undergoing elective gynecology surgery in low-resource settings.

The primary endpoints of this clinical trial were to determine the failure of the adapted OFA protocol, intraoperative isoflurane consumption and the occurrence of intraoperative complications. We found this adapted OFA protocol had a 100% success rate with significant lesser consumption of isoflurane, no significant intraoperative complication and better intraoperative hemodynamic stability in the OFA group. These findings re-iterate previous reports from other clinical trials on gynecological surgery [26, 28–31, 35–41] demonstrating a 100% success rate for OFA with stable or better hemodynamics and no intra-operative complications. By contrast, a multicentre French clinical trial on 314 non-cardiac surgeries (with 5.7% gynecological surgical procedures) reported more hypertension, hypotension and severe bradycardia (less than 45 beats/min) during OFA which prompted the early termination of the clinical trial [25]. Interestingly, all five cases of severe bradycardia occurred in prostatectomy, gastrectomy and pancreatic surgery and none in gynecological surgical procedures. Moreover, this profound bradycardia occurred at a high dose of dexmedetomidine at 0.4 – 1.4 ug/kg/h which more than doubled dexmedetomidine doses of 0.2– 0. 6ug/kg/h [30, 35, 37, 38] used by other clinical trials on gynecology surgery where stable hemodynamics were observed. Although dexmedetomidine is the reference OFA drug due to its α2-adrenergic analgesic-sedative-hypnotic-sympatholytic properties, its worldwide use is limited by its availability and relatively high financial cost. As such, adapted OFA regimens not containing dexmedetomidine such as ketamine + lidocaine + profofol + atracurium [29] or ketamine + propofol + magnesium sulfate + clonidine + rocuronium [40] or ketamine + lidocaine + dexamethasone + magnesium sulphate + clonidine + rocuronium like in the present study have been used in gynecology surgery with stable peri-operative hemodynamic profiles and no intra-operative complications. This can be explained by the fact that dexmedetomidine is a selective α2-adrenoreceptor agonist with marked dose-dependent cardiovascular depressing effects [42].

With regards to the secondary endpoints of this study, we found statistically significantly lesser postoperative pain during the first 24 h post-operation in the OFA group compared to the CGA group. This was justified by no patient requiring morphine for postoperative pain relief in the OFA group (0% vs. 33.3%; *p* = 0.0095). This can be explained by the fact that the synergistic analgesic effects of lidocaine [43, 44], dexamethasone, magnesium sulphate [45], clonidine [46] and low-dose ketamine [47] have been shown to have an overall greater analgesic effect as well as an opioid-sparing property, an anti-inflammatory effect and an anti-hyperalgesic effect than opioids. Our results concur with those of clinical studies on similar abdominal gynecological surgical procedures [29, 35], laparocopic gynecological surgery [30, 41] and mastectomies [28, 31, 39, 40] where significantly less postoperative pain, a net reduction of postoperative opioid consumption and less rescue consumption of non-opioid analgesics like paracetamol, tramadol, pethidine and ketorolac. It is worth mentioning that despite our relatively small sample size statistically significant differences in pain scores were still observed at 12 h and 24 h post-operation mainly in patients who underwent mastectomy considering the severity in pain intensity of the latter. This finding of statistically significant differences in pain intensity at 12 h and 24 h post-operation concurs with those of Di Benedetto P et al. [40], Tripathy S et al. [28] and El-dein Aboalsoud RAH et al. [31] on OFA *vs.* CGA for mastectomy in breast cancer with a similarly healthy ASA I and II small-sample population. In the same vein, the advantage of ketamine in procuring intra-operative and postoperative analgesia in OFA needs to particularly be spelled out. Ketamine analgesia is obtained from its inhibitory central and peripheral action on N-methyl-D-aspartic acid (NMDA) receptors involved in the transmission and modulation of acute pain. In addition, ketamine used at low doses in OFA has an anti-inflammatory effect and anti-hyperalgesic effects exerted still via NMDA receptor antagonism leading to good analgesia against both acute and chronic surgical pain [48]. Due to these aforementioned effects, it is quite clear now that opioids intraoperative use can be substituted by the intraoperative use of ketamine (even without dexmedetomidine as an adjunct) in similar abdominal gynecology surgical procedures [29] and mastectomy [40] like in the current study. Compared to the OFA group, the CGA had statistically significant more postoperative hypoxemia during the 1^st^ six hours warranting supplementary oxygen therapy (5.6% *vs.* 66.7%; *p* = 0.0001). We attribute this finding to the lack of opioids in the OFA group which have a respiratory depressing property compared to ketamine used in the adapted OFA protocol which has a bronchodilator effect and no respiratory depression effect [49]. This observation corroborates with those of previous clinical trials by El-dein Aboalsoud et al. [31]. on modified radical mastectomy with axillary for breast cancer surgery. By contrast, in the POFA trial on non-cardiac surgeries including 11.4% gynecological surgical procedures, more postoperative hypoxemia was observed in the OFA group (75% *vs.* 61%, *p* = 0.030) perhaps due to the additive sedative effects of dexmedetomidine, ketamine, propofol on the incidence of postoperative respiratory distress.

Opioids are potent emetics via their direct action on chemo-trigger zone receptors in the brainstem [50]. The result of this central stimulation during the perioperative period leads to PONV, more common in major gynecological surgery, where PONV occurs at incidences of 50—80% [22, 23]. Hence, similar to previous studies on gynecology surgery [28–30, 36–38, 40, 41] the frequency of episodes of PONV was reduced in the OFA group (*p* = 0.0137).

Lower rates of POI were seen in the OFA group compared with the CGA group (5.6% vs. 83.3%; *p* < 0.001) and corroborates with findings on abdominal gynecological surgery [29] and laparoscopic gynecology surgery [41]. This was an expected finding that has been correlated with the pharmacodynamic properties of opioids on the intestines: a reduction of peristaltic movements [8]. Similar to other authors working on both abdominal and laparoscopic gynecological surgery [29, 30, 37, 38] as well as mastectomy [28, 31] patients in the OFA group demonstrated statistically significant quicker postoperative mobilization or recovery with better postoperative satisfaction assessed on either the QoR-40 questionnaire or verbal rating scale for satisfaction. This reinforces evidence of OFA as being safe and effective for gynecological surgery. As an expected side effect of opioids, there were more cases of pruritus in the CGA group (6 vs. 0 cases; *p* = 0.0095). The mean cost of anesthetic drugs was lesser in the OFA group (46,500 ± 2,573 XAF vs. 57,000 ± 5,379 XAF; *p* < 0.001) as showcased in Table 4, hence, relatively cheaper and economic for resource-limited settings. No statistically significant difference was seen with regards to the mean length of hospital stay because it was a standard protocol to discharge all postsurgical women with an uneventful postoperative course on day 05 post-operation. Overall, the benefits of this adapted OFA protocol cannot be overemphasized due to a lessened economic burden on patients in resource-constrained settings but also due to the current global anesthesia era which promotes ERAS in gynecology surgery [10].

There are some limitations to the present study. Firstly, its relatively small sample size (*n*= 36) implicates the cautious generalizability of results to other low-income settings. The main reason for this relatively small size over a wide enrollment period was due to the prevailing COVID-19 pandemic which reduced the amount of elective gynecological surgical procedures performed at the study setting because of COVID-19 preventive motives. Secondly, potential advantages of OFA such postoperative opioid sparing in the geriatric population [51] and lesser respiratory depression in patients with pre-existing lung diseases [52] were not assessed. This is because the scope of study participants was limited to ASA I and II patients for the security or safety purpose of the pilot nature of this randomized controlled trial, the first of its kind in a resource-limited setting. By applying this eligibility criterion, the study population retained without any intentional selection bias were relatively young (mean age: 40.3 ± 10.7 years) and healthy patients without pulmonary pathologies, precluding the assessment of the aforementioned potential advantages of OFA. Several authors [29, 30, 38] working on OFA in gynecological surgery equally had a relatively young healthy ASA I and II study population as ours. Thirdly, the randomization of patients was more of a pragmatic approach, hence, might have flawed the study findings with allocation bias. The strength of the study relies on its rebost methods using a clinical trial with adequate patient follow-up up to four weeks post-operation to provide high-quality evidence on the efficacy and safety of OFA over CGA. Also, this proposed adapted OFA protocol not containing dexmedetomidine makes it simple, practical and economical to implement in resource-constrained environments.

## Conclusion

The current study findings suggest that the use of this adapted OFA protocol as an anesthetic technique in low-resource environments could have a promising success rate and mild hemodynamic changes with fewer observed perioperative complications when compared with CGA. In addition, the use of this adapted OFA regimen could be a safe and promising anesthetic technique/regimen to decrease postoperative complications, pain, and opioid consumption in patients undergoing elective gynecology surgery in resource-constraint settings.

## Supplementary Information

**Additional file 1.** Quality of Recovery-40 (QoR-40) questionnaire for evaluation of patient’s satisfaction.

## Data Availability

All data generated or analysed during this study are included in this published article.

## Abbreviations

ASA: American Society Of Anesthesiology
CGA: Conventional General Anesthesia
ERAS: Enhanced Recovery After Surgery
ETCO_2_: End Tidal Volume Of Carbon-dioxide
ICU: Intensive Care Unit
IV: Intravenous
LOS: Length of Stay
MAC: Mean Alveolar Concentration
NMDA: N-methyl-D-aspartic acid
NRS: Numerical Rating Scale
PACU: Post-anesthesia Care Unit
POI: Postoperative Ileus
PONV: Postoperative Nausea and Vomiting
OFA: Opioid-Free Anesthesia
YGOPH: Yaoundé Gyneco-Obstetric and Pediatric Hospital
